# “Your mind doesn’t have room for anything else”: a qualitative study of perceptions of cognitive functioning during and after recovery from anorexia nervosa

**DOI:** 10.1186/s40337-022-00723-6

**Published:** 2022-12-27

**Authors:** Johanna Louise Keeler, Carol Yael Konyn, Janet Treasure, Valentina Cardi, Hubertus Himmerich, Kate Tchanturia, Hazel Mycroft

**Affiliations:** 1 Department of Psychological Medicine, Institute of Psychiatry, Psychology & Neuroscience, King’s College London, 103 Denmark Hill, Section of Eating Disorders, London, SE5 8AF UK; 2 Department of Social, Genetic & Developmental Psychiatry, Institute of Psychiatry, Psychology & Neuroscience, King’s College London, London, SE5 8AF UK; 3 Bethlem Royal Hospital, South London and Maudsley NHS Foundation Trust, Monks Orchard Road, Beckenham, Kent, BR3 3BX UK; 4 Illia State University, Tbilisi, Georgia; 5 Department of General Psychology, University of Padova, Padua, Italy; 6 Psychological Set Research and Correction Center, Tbilisi State Medical University, Tbilisi, Georgia; 7 School of Psychology, University of Exeter, Exeter, Devon, UK

**Keywords:** Anorexia nervosa, Cognition, Interview, Qualitative, Recovery

## Abstract

**Background:**

Past research has indicated the presence of cognitive difficulties in individuals with anorexia nervosa (AN), although it is unclear how these are experienced in real life. Moreover, it is unclear how and whether the experience of cognitive difficulties changes in nature and intensity over the course of the illness and following recovery.

**Methods:**

Twenty-one female participants (AN = 11; recovered AN = 10) participated in online semi-structured interviews, utilising open-ended questions and exploring topics relating to their experiences of their cognitive function, changes over time and their perspectives on the future. Reflexive thematic analysis was conducted on the resulting transcripts.

**Results:**

Six themes were identified, centred around the effects of the illness on mental and physical function, cognition, adaptation to living with the illness, similarities between AN and other psychopathology, tentative optimism for the future and recovery as a representation of liberation. Whilst respondents with AN appeared to perform remarkably well in their professional and educational lives, the cognitive difficulties were described as significantly impacting their ability to engage in life, particularly in the context of leisure and socialising. Respondents recovered from AN noted the importance of developing a non-AN identity as well as better emotion-regulation skills as central to recovery.

**Conclusions:**

Whilst people with AN may be able to adapt their lives to the demands of the illness, they report significant cognitive difficulties that interfere with their engagement in many aspects of daily life. This should be considered by professionals working in the clinical management of AN. Treatments focused on ameliorating cognitive difficulties, and promoting emotional regulation and identity in AN are warranted.

**Supplementary Information:**

The online version contains supplementary material available at 10.1186/s40337-022-00723-6.

## Plain English summary

Individuals with anorexia nervosa (AN), an eating disorder associated with low weight, often experience poor quality of life and difficulties in thinking and brain function. In this study, 11 individuals with AN and 10 individuals recovered from AN were interviewed in order to explore the nature of these cognitive difficulties. Overall, respondents reported experiences of poor brain function, as well as similarities between AN and other psychiatric illnesses, and described how they adapted their lives around these experiences. They also were somewhat optimistic about the future, and respondents viewed feelings of liberation as a central aspect of recovery. Whilst individuals with AN often appear to be functioning very well, it appears that there are several difficulties associated with brain function and mood that may interfere with the ability to engage in daily life, especially in leisure and social activities.

**Supplementary Information:**

The online version contains supplementary material available at 10.1186/s40337-022-00723-6.

## Introduction

Anorexia nervosa (AN) is an eating disorder characterised by significantly low body weight, an intense fear of gaining weight or fatness, disturbance in body perception and severe restriction of food intake or other measures to lose weight leading to significantly low weight [1]. Prognosis is poor, with one study showing almost two thirds of participants remaining unwell at a 9-year follow-up and one third remaining unwell at 22 years [2]. Illness of this length is often referred to as “severe and enduring”, and there is emerging evidence that physical sequalae are likely to contribute to functional difficulties as the illness progresses [3]. Currently, the factors that contribute to the maintenance and perpetuation of AN are unclear, although these may be related to brain changes [3, 4], metabolic and hormonal changes such as low leptin levels [5] as well as the presence of psychiatric comorbidity either preceding or succeeding the illness. Psychiatric comorbidity is common in AN, with depressive and anxiety disorders and symptoms being common in approximately 50% of patients [6, 7] and autism spectrum disorder in up to 35% [8]. The loss of brain volume and presence of comorbidity is likely to contribute to functional difficulties, including problems with cognitive functioning and social cognition [3, 9]. Whilst this has been explored to some extent in the literature, there has been less attention given to the consequences of alterations in cognition on quality of life, daily functioning and illness progression in people living with AN.

The evidence for cognitive difficulties in the acute stages of AN is largely centred around challenges with cognitive flexibility [10] and “bigger picture thinking” (i.e. central coherence; [11]), although there is also evidence for negative biases in attention [12] and memory recall [13], as well as generalised problems with decision-making [14] and memory [15]. Indeed, in a recent meta-analysis, memory problems were found to be the measure of neuropsychology diminished the most in adults with AN [16], although the extent and nature of these memory problems is uncertain (e.g. are they more akin to neurodegenerative diseases, or to depressive disorders, for example). Neuropsychological research often uses a threshold of “cognitive impairment” of two standard deviations below the normative score [17], or reports a statistically significant difference between patients and unaffected controls. However, it is unclear how this inefficiency translates to a subjective experience of impaired function in real life. A prior study in depression has shown a lack of association between objective and subjective assessments of cognitive impairment, and the propensity to experience objective cognitive impairment was increased with the severity and duration of depression as well as younger age [18]. This exemplifies a discrepancy between the two forms of assessment [19].

There has been little research into subjective cognitive complaints in AN, which may contribute to the poor quality of life experienced by people with both acute and chronic AN [20]. Such research may help to elucidate factors that both maintain the illness and facilitate the transition towards recovery. As noted by Feyaerts and colleagues, the way to unite the subjective experience of psychiatric symptoms with empirical findings, is to “return to the things themselves” [21, 22]. A research phenomenological meta-synthesis conducted by Bryant and colleagues [23], incorporating data from 1,557 people with AN, highlighted the role of restrictive eating in mitigating difficult and confusing emotions to aid the individual in navigating a threatening world, as well as providing a sense of self and identity. The authors remarked on the lack of qualitative data on cognitive difficulties and traits associated with AN, and how this impedes interpretive potential [23]. Thus, although functional impairment is identified as a sub-theme in this study, the studies available for the synthesis did not enable a more specific exploration of subjective cognitive function in AN. Notably, in the investigation of cognitive function associated with AN, it is important to explore how people who have recovered from AN experience these difficulties. People with acute AN often display poor insight into their illness, which is usually noticed by peers and clinicians rather than the individuals themselves [24, 25].

Given people with AN report poor quality of life, exploring the nature and intensity of functional problems may elucidate new targets for treatment. In line with this approach, adopting people with life experience of AN as “experts by experience” provides the opportunity to identify which aspects of the illness are the most prominent or are contributing to functional difficulties. Moreover, having insight from individuals recovered from AN may help to identify aspects that facilitate recovery and thus could be targeted by treatment. The aim of this qualitative study was to explore participants’ subjective experiences of cognition, perspectives on the future, and how these change over the course of AN and after recovery.

## Materials and methods

### Participants

Twenty-one female participants (AN = 11, rec-AN = 10) who previously took part in an online neuropsychological study of autobiographical memory and future thinking [26] were recruited in this follow-up study. Inclusion criteria for all participants included the following: age ≥ 18 years, fluency in the English language, access to a computer and stable internet connection. Participants were excluded if they had a history of or current post-traumatic stress disorder, substance abuse or psychotic disorder. Participants with AN had to have a self-reported diagnosis from a clinician according to the Diagnostic and Statistical Manual of Mental Disorders—5th edition (DSM-5; [1]) or International Classification of Diseases 10 (ICD-10; [27]) and a body mass index (BMI) of ≤ 18.5 kg/m^2^. Participants in the rec-AN group had to have a self-reported historical diagnosis of AN from a clinician, with no self-reported behavioural or psychological symptoms and a BMI of ≥ 18.5 kg/m^2^ for ≥ 1 year.

### Design

Semi-structured interviews were conducted by the lead researcher (J.L.K.), which lasted for up to an hour. The interview started with an overview of the participants journey with AN, and then consisted of three sections: their experiences of their cognitive functioning; changes over time; and perspectives on the future (see Additional file 1 for a full interview schedule). The researcher used prompts to prime further elaboration. Participants were also invited to discuss their eating disorder more broadly and to discuss any other important aspects that were missed during the interview schedule.

### Measures

#### Demographics

A bespoke demographic questionnaire measured the following variables: age, gender, ethnicity, years of education, highest level of education obtained and medication usage (the latter four measures not reported in this study). Self-reported weight and height was recorded, which was used to calculate BMI in kg/m^2^.

#### Eating disorder psychopathology and characteristics

The Eating Disorder Examination-Questionnaire [28] was used to quantify eating disorder psychopathology (Cronbach’s alpha = 0.97). The EDE-Q is a 28-item self-report questionnaire that assessed the severity of and features associated with eating disorders, and constitutes four subscales (Eating Concern, Shape Concern, Weight Concern, Restraint), which are averaged to provide a Global score. Participants in the recAN and AN groups were asked to estimate their lowest BMI, as well as the duration of their eating disorder diagnosis and symptoms (in years) and their current treatment. Participants in the recAN group were asked to estimate how long they had been recovered for (in years).

#### Comorbidities

Participants were asked to self-report whether they have received a diagnosis of a comorbid psychiatric disorder, including mood disorders, anxiety disorders, personality disorders or any others. Symptoms of depression and stress were measured using the Depression, Anxiety and Stress Scale-21 (DASS-21) [29], which is a 21-item self-report questionnaire assessing depression, anxiety and stress (7 items each) over the previous week using a series of statements and responses ranging from 0 (“Did not apply to me at all) to 3 (“Applied to me very much or most of the time”). The Cronbach’s alpha for the DASS-21 was 0.95.

### Procedure

Following recruitment of the AN and rec-AN samples for the prior study [26], all participants who consented to being contacted for future studies were emailed details of this follow-up interview study. Those who replied were provided with an information sheet and consent form for the study. Interviews took place online over Zoom, which were audio recorded and automatically transcribed. Transcripts were verified and modified against the audio recordings by researchers (C.Y.K. and J.L.K.), and participants’ personal details were removed from transcripts to preserve anonymity. All recordings of interviews were deleted after the transcripts were verified for accuracy.

### Data analysis

Transcripts were imported into and analysed using NVivo 12 [30]. Reflexive Thematic Analysis in its six stages, as defined by Braun and Clarke [31], was used to analyse the qualitative data. Two independent researchers (J.L.K. and C.Y.K.) familiarised themselves with the data through several reviews of the transcripts. Initial coding was independent and occurred through line-by-line reading of the transcripts. Discrepancies between initial codes were discussed and the conceptualisation and coding of labels was revised once a consensus was reached. A third investigator (H.M.) was consulted in the case of disagreements. Codes were then analysed further to identify themes and subthemes within a thematic model. The identification of themes and subthemes occurred in an iterative fashion, involving the three aforementioned researchers, with acknowledgment that within qualitative analysis there is no one ‘right’ answer [32].

Within the thematic analytical framework, we initially sought to identify participants’ broad experiences of cognition and future thinking before, during and after experiencing AN. The initial stages of the analysis process, and in particular the structuring of initial codes into themes and subthemes, was partly deductive in that it aligned with the current literature on cognitive function within AN. Following this, we attempted to keep the topic and analytical process broad and inductive to capture participants’ wider experiences of living with AN and thereby potentially identify any other potentially relevant and/or meaningful aspects of experience. Throughout the entire process, all researchers considered how their prior experiences and knowledge may have impacts on the research (see the authors’ reflexive positionings for contextual information on their respective experiences and research interests).

## Results

### Participant characteristics

The demographic and clinical characteristics of the participants in the AN and rec-AN groups are presented in Table 1. Participant age ranged from 20 to 43 (M ± SD = 27.1 ± 6.2). Nineteen participants reported their AN subtype, most of which was the restrictive subtype of AN (n = 17) with two participants having the binge-purge subtype of AN. The current BMI in the AN group was 14.8 ± 1.9 kg/m^2^ and in the rec-AN group was 20.3 ± 2.1 kg/m^2^. Diagnosis duration in the AN group ranged from 0.1 to 21.1 years (M ± SD = 10.2 ± 6.2) and from 3 to 11.5 years in the rec-AN group (M ± SD = 9.7 ± 3.0). Recovery duration was reported by eight participants in the rec-AN group, which had a M ± SD of 2.6 ± 1.7 years. In terms of eating disorder psychopathology, the average EDE-Q Global score in the AN group was 3.8 ± 1.0 and in the rec-AN group was 1.1 ± 0.9. Depression and anxiety were the most commonly reported comorbid psychiatric diagnoses, reported by 12 and 9 participants, respectively.

**Table 1:** Demographics and clinical characteristics of participants

Participant	Age	AN sub-type	Current BMI (kg/m^2^)	Lowest BMI (kg/m^2^)	Co-morbidities	Current therapy	EDE-Q Global Score	DASS Overall Score	Duration of symptoms (years)	Duration of diagnosis (years)	Duration of recovery (years)
*Current anorexia nervosa*											
P01	23	–	17.2	16.4	Anxiety Depression	None	3.4	48	7	3	N/A
P02	29	AN-R	10.8	10.3	Anxiety	Inpatient	3.3	90	15	8	N/A
P03	43	AN-R	15.2	14.7	None	Outpatient (Private)	2.4	54	12	14.5	N/A
P04	30	AN-R	12.8	12.2	None	Outpatient	4.8	84	22	16	N/A
P05	25	AN-R	13.4	9.7	Depression EUPD	Outpatient	3.4	70	14	13	N/A
P06	31	–	17.3	12.0	Depression	Outpatient	5.5	104	20	9.8	N/A
P07	25	AN-R	14.5	10.7	Anxiety Depression	None	4.1	40	11	10	N/A
P08	23	AN-R	13.7	13.7	Depression	Outpatient	2.6	88	12	13	N/A
P09	20	AN-R	15.9	15.7	Anxiety Depression	Outpatient	5.3	70	12	0.1	N/A
P10	34	AN-BP	15.5	14.6	Depression	None	4.0	96	20	21	N/A
P11	32	AN-R	16.9	15.1	Depression	Outpatient (Private)	3.2	8	4	4	N/A
*Recovered from anorexia nervosa*											
P12	29	AN-R	19.4	16.9	Anxiety	None	1.8	20	10	7	3.5
P13	33	AN-R	19.0	13.2	Anxiety ASD	None	0.4	6	13	9	5
P14	21	AN-R	19.0	14.4	Anxiety Depression	None	0.2	26	5	3	1.1
P15	23	AN-R	25.2	16.9	Depression	–	2.7	12	9	4.3	-
P16	25	AN-R	19.4	14.9	PTSD	–	0.5	8	12	9	5
P17	23	AN-R	20.5	13.8	None	None	0.5	26	10.9	9.3	1
P18	20	AN-R	18.8	14.0	Anxiety Depression	–	1.5	20	5	3.3	2
P19	29	AN-R	19.8	13.0	Anxiety Depression	None	0.9	36	13.3	11.5	-
P20	20	AN-BP	22.8	18.6	None	None	2.3	56	8	4	2
P21	21	AN-R	19.0	14.5	None	–	0.5	26	11	10	1.1

*AN*  anorexia nervosa, *AN-BP*  AN binge-purge subtype, *AN-R*  AN restrictive subtype, *ASD*  Autism Spectrum Disorder, *BMI*  body mass index, *DASS*  Depression, Anxiety and Stress Scale, *EDE-Q*  Eating Disorder Examination-Questionnaire, *EUPD*  Emotionally Unstable Personality Disorder, *PTSD*  Post-Traumatic Stress Disorder

### Qualitative results

**Fig. 1:**
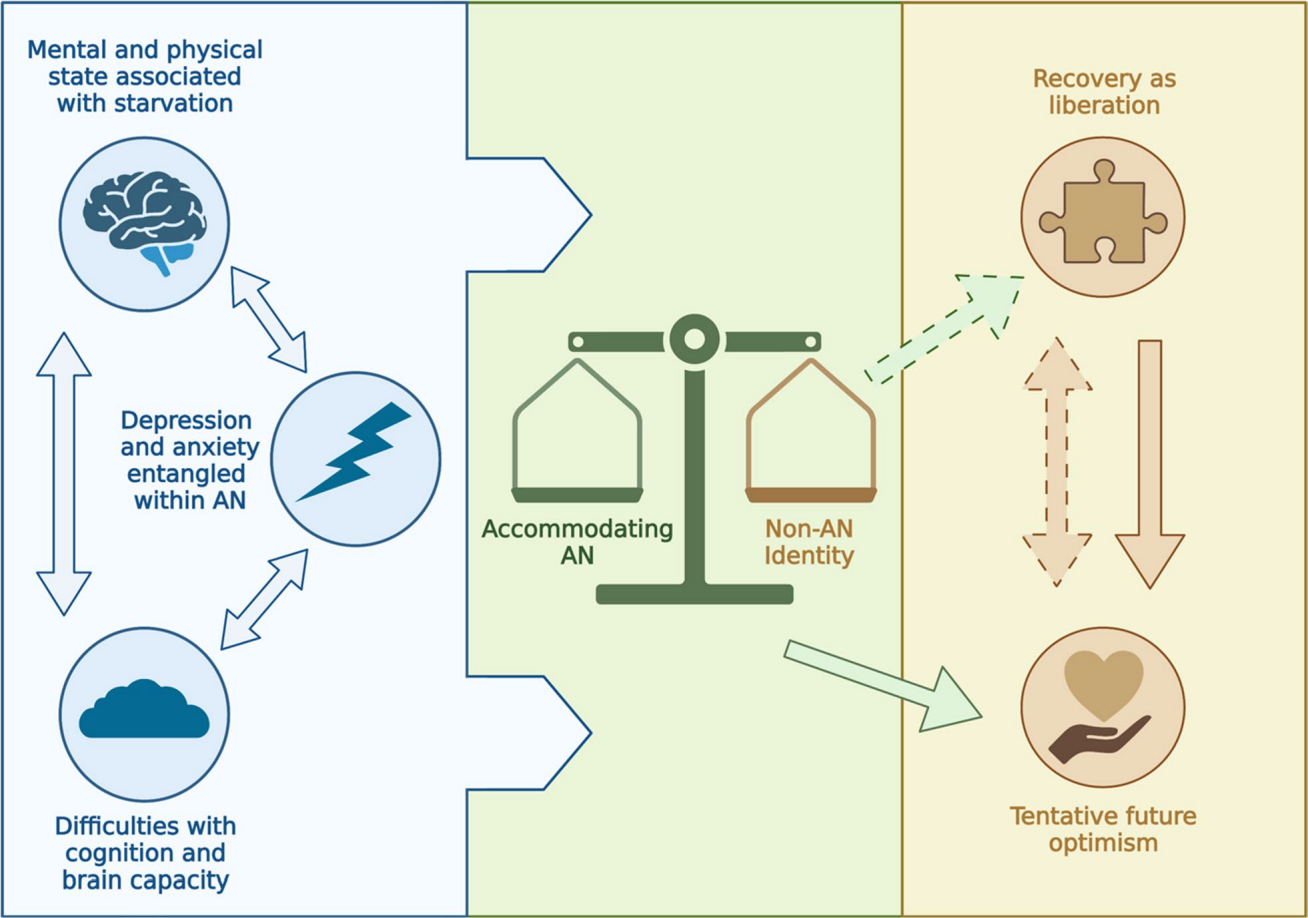
Diagram depicting how the acute experiences of depression and anxiety, problems with cognition and effects of the illness on the brain and body within AN are interlinked. These acute experiences directly contribute to the conflict between accommodating AN, in terms of daily life, and developing a non-AN identity, which may ameliorate some of the acute cognitive and affective difficulties. The development of a non-AN identity, in contrast to accommodating to AN, may lead to recovery and liberation (represented by the dashed green arrow). This in turn improves optimism for the future, which sustains recovery (represented by the bidirectional dashed amber arrow). On the other hand, accommodating to AN led to positive views of the future, albeit these perceptions were biased towards AN (represented by the non-dashed green arrow). People acutely unwell with AN were able to see recovery as liberation, leading to a positive future. However, the inability to perceive a future with AN obstructed thoughts of recovery (represented by the single-sided amber arrow), leading to feelings of stuckness and hopelessness. Double-sided arrows represent a bidirectional relationship whereas single-sided arrows represent a potentially causative pathway. Created with biorender.com

Six key themes were identified following our analysis of the AN and rec-AN interviews, which are depicted in Fig. 1, overviewed in Table 2 and explored in greater detail in the following sections.

1. Poor mental and physical function associated with starvation
2. Difficulties relating to cognition and brain capacity
3. Accommodating AN
4. Depression and anxiety entangled within AN
5. Tentative optimism about the future
6. Recovery representing liberation

**Table 2:** Overview of themes, subthemes and example quotations

Theme/subtheme	Quotation
**Poor mental and physical state associated with starvation**	“And I think, with time everything's generally got a bit harder, I think, early on my body tolerated not eating enough and being at a low weight better than it would do now.” (P06)
*Physical consequences*	“Like my health conditions, like physically I was feeling absolutely like awful because through the years, like four or five years, I had this condition, um, my health drastically… drastically became worse.” (P20)
*Brain function*	“…I really do feel like I've lost some part of. Like working part of my brain, I really do feel like that…” (P01)
**Difficulties relating to cognition and brain capacity**	“But when I’m in a relapse, my brain is not working, at all, very well.” (P10)
*Poor information processing* Autopilot Attentional bias Alterations in memory	“when I had anorexia I just remember, like people speaking and I just be like trying to like comprehend their words and just trying to like piece it all together and like… it was like a real effort to make sense” (P13)
*Overcontrolled and rigid repertoire*	“it's obviously quite irrational so sometimes if I had certain routines or things I had to do, I’d have to do them first before I could do anything else” (P02)
**Accommodating AN**	“I mean in a way it hasn't, I've adapted so that I am able to live my life alongside it.” (P02)
*A narrowed focus*	“You don't value anything else. Um really, because that is like the main priority, is losing weight and so when you don't value anything else, you don't attribute meaning to anything else.” (P12)
*Salient anorexic identity*	“Sometimes I feel like when I’m labelled as having an eating disorder and put in inpatient or institutions, that’s when it flares up because then that’s suddenly all I am, all I have.” (P02)
*High achievements despite personal sacrifice*	“I still kind of got good grades, first year of university, I did a lot better than I have since then because I wasn't really doing anything else.” (P14)
**Depression and anxiety entangled within AN**	“My obsessive thoughts get worse, and my mood, if I'm at a very low weight, my body struggling, my mood can get worse as well” (P05)
*Depression as an antecedent and consequence*	“Although sometimes there's, a point which, if my weight is going down. That can alleviate my mood which enables me to be more flexible… which is where the difficulties come in, because I mean that, that happy spot doesn't last very long.” (P06)
*Dichotomy between emotional reactivity and blunting*	“…I've become like obviously not fuelling myself properly, so I think you just become way more sensitive and more emotional but also numb at the same time, if that makes sense.” (P01)
**Tentative optimism about the future**	“I'm not sure it [AN] will ever fully go away, but.. yeah, as I said, having a better quality of life than I have now” (P08)
**Recovery representing liberation**	“…it's being able to. To run again and to exercise but also not compulsively and the ability to maintain a job. And although I do like my routine maybe to be a bit more flexible, so I can say yes to things more.” (P08)
*Identity as liberation*	“…at the time I was like, I don’t have a favourite colour, I don't have a favourite book, I don’t have any hobby, like, I'm-. I just felt like a shell, whereas now I feel so developed, like, I know who I am and what I like.” (P16)

As seen in Fig. 1, the identified themes and subthemes form an overall thematic framework that broadly represents participants’ experiences of living with, and recovering from, AN. Three themes were distinct but interrelated with bidirectional effects: the effects of starvation on the brain and body, the depression and anxiety associated with AN, and cognitive difficulties associated with AN. In particular, difficulties relating to cognition and brain capacity both perpetuated and were perpetuated by depression and anxiety, and contributed to (and were exacerbated by) a poor mental and physical state. People with AN adapted their lives and identities to these difficulties. This accommodation acted as a maintaining factor, as respondents’ perspectives of the future were positive, but broadly biased towards AN. However, this accommodation also made engagement in important daily activities (e.g. social interactions) difficult, due to an incompatibility between the demands of AN and the demands of external forces. In this context, respondents with AN recognised recovery as a route towards liberation and freedom. Liberation represented freedom from the physical and cognitive aspects of AN (blue section) and the subsequent effects on identity and quality of life (green section). On the other side of the scale, those recovered from AN were able to develop and nurture an identity not related to AN. These respondents had experienced recovery as liberation and thus had optimistic perceptions of the future. This fed back into perceptions of recovery as liberation; recovered respondents identified the need to avoid relapse and sustain this liberation in order to achieve their future goals. Similarly, perspectives on recovery and its representation as liberation were restricted by negative thoughts of the future; participants with AN, particularly who could not imagine an identity without AN, either saw things as continuing as the same or were biased towards AN being integral in their futures.

## Poor mental and physical state associated with starvation

### Physical consequences

Participants reported physical consequences of AN, which they attributed to prolonged starvation and low weight. Respondents reported that these physical consequences (e.g. coldness) had widespread implications for mood, cognitive function (e.g. concentration), and the ability to engage in everyday life.

*“Being always like really cold because I was underweight and... I remember like being so kind of fixated on this like being cold like it would just overwhelm me, so yeah.”* (P19)

Physical exhaustion was mentioned by several participants, which interfered with their ability to engage in education, work and social interactions. This exhaustion was attributed to brain overdrive and was reported to contribute to problems with concentration and brain function. One participant reported this akin to the physical and mental tiredness similar to the day after a poor night of sleep.

*“obviously doing everyday activities was really difficult because I was just so tired and didn't have any energy... and, and I guess it was more a case of trying to... figure out how I was going to get through the day”* (P21)

### Brain function

During the acute phases of illness, problems with brain functioning were noted, including “fuzziness” and “fogginess” in thinking, which was ameliorated by weight gain and intensified by further weight loss. In particular, several respondents suggested that poor brain function may be related to the body being in “survival mode”.

*“My thought process- like I just feel like my brain wasn't going to be like helping me remember stuff it was like focused on survival is what I think and like... I learned also everyday be in the same so like I haven't got one moment that I could remember but yeah I just felt like my brain was not functioning at its... full potential, because now my brain works really well, but like back then like no it did not.”* (P13)

*“The lack of functioning of my brain like I really do feel like I've lost some part of... like working part of my brain, I really do feel like that, I don't even think it's the, I mean it could be partly the hyper focus on food that’s taking up the majority of my brain, like power. But I also think it's probably just because I haven't- I'm malnourished in that sense.”* (P01)

Others attributed problems with brain function to the fatigue and tiredness associated with malnutrition.

*“yeah and I think what affects my thinking and cognition more now is the tiredness and the fatigue and malnutrition, rather than the thoughts about food.”* (P06)

## Difficulties relating to cognition and brain capacity

Participants identified a host of cognitive features associated with AN, which were related to: poor information processing, an overcontrolled and rigid repertoire, and a tendency for forward planning in order to manage uncertainty. Often these features resulted in distinct problems with function and with self-perception; several participants described how cognitive difficulties fed into their core beliefs of themselves as incapable, rather than them attributing difficulties to the illness.

### Poor information processing

#### Autopilot

Many of the recovered respondents described feelings of automaticity and monotony in daily life during the most severe phases of their illness. This was characterised by a feeling of mental numbness, fogginess and fuzziness towards external events and stimuli.

*“At the worst of it, I don't think I, I think I was just living really not, or sorry, just like sort of existing, I don't know, nothing from that period to sticks out on when I try and think about it, I think I, so it's just so hard to think of like... what the day-to-day life was because every day felt exactly the same”* (P18)

#### Attentional bias

Almost all respondents described a narrowing of their thought patterns as they became heavily biased towards AN-related topics, such as food, weight, calories and shape. These topics were described as taking priority over everything else in life within the mental headspace. Thought patterns were described as relentless, repetitive and restricted to specific topics. Feelings of frustration were described as some respondents had insight into how this headspace could be used for something else, although they felt powerless to redirect their thoughts elsewhere. Often respondents connected this to feelings of isolation, as a by-product of an inability to connect with the external world (e.g. education, work, social interactions).

“*It kind of is in a similar vein to just the very idea that your brain is so taken up with thoughts of food, thoughts of eating, thoughts of calories, thoughts of wanting to be thin and what you ate, what you're going to eat, what you didn't eat... That it's just like your mind doesn't have room for anything else. And I really wanted to have that room, I could recognise I didn't have room for anything else, but I wanted to, and that was really frustrating”* (P12)

Moreover, several respondents noted the effects of this attentional bias on memory encoding. As AN-related information, stimuli and contexts became the priority in attention, other content was not paid attention to and therefore not encoded into memory.

*“...the main priority, is losing weight and so when you don't value anything else, you don't attribute meaning to anything else. I can see why it would stay in your brain, I think your brain remembers important things, interesting things, things that are different, and when you're not interested in anything else you're not going to remember anything else.”* (P12)

#### Alterations in memory

Overall, many respondents described distinct difficulties with memory, some of which were more common across the group (e.g. negatively-biased memory recall, poor overall episodic memory but good memory for notable events) and some of which were less common (e.g. poor visuospatial memory; poor prospective memory). A few of these participants, all of whom had severe-enduring illness (≥ 9 years) noticed the problems worsening throughout the course of their illness. However, several other respondents could not identify any problems with their memory.

##### Biased encoding and memory recall

Several respondents noticed a bias in their memory recall, particularly towards disorder-related or negative information, both for recent memories and when recalling the AN period in general. Some considered that an attentional bias towards negative and disorder-related content during encoding may be driving their biased memory recall.

*“I think I definitely would remember like what I had eaten, for every meal and for every snack, for like the last week in vivid detail. And, but I'm not sure other than that. I think a lot of my brain space starting going to remembering that.”* (P14)

##### Poor overall episodic memory but good memory for notable events

Respondents who had recovered from AN described a period of amnesia, in not being able to remember details of their unwell period. However, they were able to recall details of specific notable events during this period, particularly those that were negative, traumatic and stressful, as well as key turning points. Several reasons for their overall poor episodic memory were proposed, for example feeling disconnected from their unwell selves, or not wanting to remember a negative period of their lives. Some could connect with the emotions they felt during the time but could not visualise details of the episode clearly.

*“it's so weird, I feel like I didn't realise like how much of it I just do not recall until having to like actively think about it, it’s really odd.”* (P18)

Participants with current AN described a general inability to remember events well, both in recent and distant times, again excluding their good memory for particularly notable events. Some identified these difficulties as arising since the AN episode began.

*“But I still have quite good memories of stuff and then as soon as it went to anorexia like I don’t- I don't really remember.... yeah from like 2021 to now like it’s very blurry.”* (P01)

Both those with acute AN and who were recovered from AN explored reasons for their good memory of notable events, which were largely centered around the event being particularly impactful on themselves. For example, events that were traumatic, stressful, or particularly pivotal to their experience of AN, elicited strong emotions and in the context of feeling emotionally blunted, are particularly notable.

*“I had such intense emotions in the moment. I think it kind of like imprinted in my mind, but I don't think I had like as many other like memories which were as intense, I did have some also, but they were just still... I think I’ll have to say, they were not as impactful on my memory if it's like, if I can say that. Like, even though I can remember them like slightly, not detailed, though.”* (P20)

##### Specific difficulties in wider memory abilities

Several participants described problems with specific forms of memory, which caused interference in their lives. Distinct difficulties with visuospatial memory were described, such as forgetting where they were supposed to be going, or forgetting where they had placed objects.

*“I remember once I had, I came into the kitchen and both my housemates were like ‘S, did you put a bag of peas back in the cupboard instead of the freezer?’ and I was like... did I? I lost it, just like strange things like that, where you just blank and, of course, at the time I was like oh God it's early onset Alzheimer’s or something, but yeah, now that I think about it, maybe it was because of what was going on.”* (P12)

*“And like when I looked at the route there, I forgot like five minutes later and had to look at the route again.”* (P07)

Other specific difficulties included forgetting words (e.g. names, places, films) and dates (i.e. nominal dysphasia). Several respondents described problems with storing information and working memory, having to go over information several times in order to remember later on.

Furthermore, problems with remembering to execute plans was described (i.e. prospective memory).

*“Oh, you know things like my mum will say, ‘can you do this for me’ and I’ll be like ‘yeah sure’, and I will forget to do it..”* (P05)

### Overcontrolled and rigid repertoire

A prominent characteristic of AN described was a dichotomous, or “black-and-white" thinking style surrounding food, academic/work performance and general life.

*“...it kind of blend into all areas of my life, like, if I believed something was right or wrong, like there was a correct way of doing things and if it, if it deviated from that way, then it was... absolutely wrong and there was no leeway in it. And like never any middle ground like everything was black or white.”* (P16)

Most rationalised this thinking style as trait perfectionism, although underlying this was a general fear of failure and an aversion to risk and uncertainty. Several respondents described how this intense perfectionism persisted even in situations that had no strong personal incentive.

*“yeah, I think I should say, maybe it's kind of that perfectionism that I think a lot of people with anorexia probably have, so it was like I wanted to achieve really good grades, but just for sake of it, more than anything.”* (P14)

This is reminiscent of a habitual quality that AN had for some participants; despite recognising the dangers of the illness, many respondents described powerlessness over its hold.

*“Even though, like in the back of my mind I understood that, like not eating is not good, I couldn't stop too because, like I’d say I saw results kind of. Like I lost weight by basically restricting almost all my food, and I just couldn't stop it was like such an addictive behaviour to me.”* (P20)

The disorder was described as prescriptive over respondents’ lives, where their days and plans were structured around a rigid framework of rules surrounding calorie intake and compensatory behaviours (e.g. exercise). This exemplifies a paradox in control whereby the anorexic tendencies are characterised by heightened control, albeit respondents described having little control over the AN compulsions. Therefore, flexibility and spontaneity could only exist within the boundaries of a pre-defined and rigid structure, in order to manage uncertainty, anxieties and anorexic compulsions.

*“...if I was going out with friends or going to a family event that involved food. Like if I knew that was coming then it would be like. I’d be thinking about what I’d do, like saying the week prior to it to kind of, allow me to engage or like eat and eat with my family or my friends, it was like, you know, I had to kind of compensate for it.”* (P19)

Several respondents described how these rigid and inflexible tendencies were more pronounced at a lower weight, as a consequence of increases in thoughts surrounding food, weight and shape and concerns about losing control.

*“And I noticed that, when my food is worse or when I’m kind of struggling with food more, I, or my weight is dropping, I do become more rigid and inflexible, but some of that is to do with if my mood is low and I’m feeling overwhelmed or there's a lot of things going on in my life and that just makes it harder for me to be flexible... And when food is difficult, that. It removes some of that stability and makes the rigid- rigidity, worse.”* (P06)

Conversely, following recovery and/or weight gain, high levels of control over these rigid tendencies were described. Overall, respondents recovered from AN described greater flexibility in their thoughts and behaviours.

*“I'm still quite rigid person, but it... there’s much more. Like I feel like I’m in control of that more like I can bend what I want to do if it’s on my own terms.”* (P16)

## Accommodating AN

### A narrowed focus

Participants reported feeling unable to enjoy daily activities due to the preoccupation with exercise, food, and weight. Some acknowledged this preoccupation as irrational and yet described how engaging in eating disorder-related behaviours provided a sense of achievement and reassurance. For most, prioritising their eating disorder led to disengaging from social interactions, and consequently social isolation.

*“I always had a difficult relationship with exercise as well it's like. If I can't exercise that's always been the priority so it's. You know, I had to kind of. Almost like- I miss- missed out on certain things because of it, or you know, compromise, that would be the word, compromise quite a lot.”* (P17)

Social avoidance was perpetuated by social anxiety, including social comparison and problems with reading social situations surrounding food. Most respondents reported not being able to eat in front of others and intense anxiety and avoidance behaviours resulting from the possibility that they may be pressured to eat, without extensive preparation in advance. The AN-related demands were often incompatible with the demands of social situations.

*“I never eat out or anything with my friends like if they do something that involves like socially eating, like I’m really scared to like go out all the time, because I’m like oh they're going to like force me to eat, they're going to judge me for how much I’m eating.”* (P07)

Several of the participants reported adapting everyday life to accommodate eating disorder-related obsessions and compulsions, and their rigid repertoire, creating a false sense of spontaneity.

*“I kind of just adapted to things so if a friend wanted to meet up that would be fine as long as I could find a route that involved the most walking then I had it covered and I was okay to, like, do something spontaneous like that.”* (P02)

### Salient anorexic identity

The AN identity developed over the illness and became more salient as the illness progressed, both in severity and duration. In respondents with current AN, this was exemplified by poor insight regarding the severity of the illness and the extent to which it had affected their cognition and ability to engage in day-to-day activities.

*“I think it hasn’t really impacted me, if I’m honest, because I haven’t really struggled to concentrate, at least with my studies.”* (P08)

This also extended to their inability to perceive the extent of their weight loss; AN participants presented difficulties with body perception and self-image.

*“I can't see a problem when I look in… when I look in the mirror, but my dad's like “are you actually insane, like you, are insane if you can't see anything wrong with you”, and people stare at me in the streets and I'm like “why are you staring at me”.* (P04)

Many of the AN participants considered their obsessive and inflexible tendencies as innate characteristics that became worse over time as a result of AN, thus cementing the idea of AN as part of the identity.

*“I think I was always, even before I got ill I would say I was probably quite rigid also because I was very, very active and I did a lot of sports and I think even as a child, if I hadn't been as organised or if my day hadn't been that scheduled, then I wouldn't have been able to do all those things, but I think it probably did get a bit worse.”* (P08)

Notably, AN participants reported perceiving weight gain and recovery from AN as a threat to their selfhood.

*“Just scared of the way I felt. I just felt [whilst weight-restored] like – I felt big, I felt uncomfortable in my own skin... I thought that people would start forgetting about me because I was like normal now.”* (P04)

### High achievements despite personal sacrifice

Although many of the participants reported several cognitive problems, most were also able to obtain high academic achievements. This was attributed to their perfectionistic tendencies, allocating their limited cognitive resources towards academic success, and exploiting studying as a means of distraction from eating, thinking about food, and hunger.

*“In terms of doing my art foundation for instance that's where I was at college last year and I did manage to complete it, and I actually got a distinction. So, even though I have this diagnosis of anorexia and you know my weight was quite low I was still able to function enough to be able to do something quite considerable.”* (P02)

Some expressed feeling disheartened by this and expressed that if they did not endure the adverse effects of AN, they would be able to apply their cognitive capacities elsewhere—beyond thinking about exercise, food, and weight.

*“My mind is focused on food far more than other people’s would be and that seems like a bit of a waste.”* (P03)

## Depression and anxiety entangled within AN

### Depression as an antecedent and consequence

Low mood was reported as a common feature of AN, which was broadly categorisable into being a consequence, or cause, of AN. However, many participants described difficulty in disentangling the two. Some respondents described a process whereby engaging in disordered eating behaviours initially alleviated negative emotions and provided a sense of achievement, which quickly led into a deep depression. The experience of this “sweet spot” before the subsequent plummeting in mood was self-reinforcing; chasing this feeling by engaging in further disordered behaviours further perpetuated the low mood, which was described as worse when at lower weights.

*“sometimes like in therapy they could be like ‘oh, do you not think that your low weight is why you feel bad’ and it's like, ‘no’, I think I felt bad before, and I used my weight because it was the thing in my life I could control when I felt bad.”* (P05)

Participants who had relapsed multiple times described how the low mood intensified at every lapse as a function of hopelessness.

*“When I notice, yeah that I go into another relapse. Yeah, each time there's a bit more despair, because you think oh gosh yeah I’ve had this before.”* (P03)

Depressive features of AN were mainly characterised by anhedonia. Respondents described how they felt emotionally numb with a restricted spectrum of emotions, related to the purpose that AN serves in blocking out overwhelming and negative emotions. Some noticed that at a low weight, their mood was lower and concurrently their rigidity was greater. Being inflexible was described as serving a purpose to minimise negative and racing thoughts and reduce the chances of low mood.

*“I think that like the anorexia. The way of like it feeling in control and feeling that it was being inflexible it's like if you're. If you, I think, almost a bit out of fear like if you, if you don't follow these rules, or you don't do X, Y, Z, then you're gonna feel awful. And this is- it’s like- if you're inflexible you're not going to feel awful so it's like a way of controlling those very out of control emotions, I’d say.”* (P17)

### Dichotomy between emotional reactivity and blunting

The majority of respondents with AN described a high level of generalised anxiety, particularly surrounding food situations and periods of change or uncertainty. Catastrophisation was described both generally and in the context of worries surrounding food—for example, the consequences of eating a feared food were both extremely negative and hyperinflated.

*“I just remember being like that, everything felt so huge in my head, like the idea of eating a few grapes, the idea of having like a bit of sugar in my coffee or whatever it was just so enormous, and I think that sticks out at the time my mum going like ‘what?!’”* (P18)

Relatedly, respondents described being irritable and emotionally reactive to stress, which several described as irrational and delusional. This was amplified when at a lower weight despite feeling emotionally numb.

*“Because I've become like obviously not fuelling myself properly, so I think you just become way more sensitive and more emotional but also numb at the same time, if that makes sense.”* (P01)

## Tentative optimism about the future

All participants were asked to describe themselves in 10 years. Responses were diverse but were centred around: images of the future being based on current or past experience; a bias towards AN; an inability to think about the future. Overall, respondents were generally optimistic about the future, although almost all participants with AN thought that they would not be recovered.

Many respondents reported that they expected little change in their life; perceptions of the future were often contingent on past experiences or the present. To some extent, this leant into a bias towards an AN-oriented future, and often respondents’ current mental health state prescribed their visions of the future (e.g. because I’m currently feeling low, the future is bad). One participant described integrating their present into the future as a coping mechanism for coming to terms with their current situation.

*“…at the minute, I don’t feel very hopeful so I see it the same or a bit worse, really.”* (P10)

Several respondents described difficulty in thinking about the future. Some felt hopeless about their future as a result of feeling hopeless about recovery. Others described not knowing what was realistic, or not knowing what their long-term goals were. Finally, some described needing to have control over their current schedule, and thus working on a week-by-week basis was incompatible with thinking about the future in terms of years ahead.

*“It is quite, It is quite, It is quite difficult, because I kind of like live in the moment and don't think too far ahead, I do like to be organised with like my plans and stuff like if someone makes plans they need to give me like a week’s notice of the plans. But I can't think as far ahead as years, like, I think, like I work on like a weekly basis.”* (P07)

Those recovered from AN were overall able to imagine the future more clearly, which was attributable to having a sense of purpose. Additionally, the absence of constraints of living with AN led to greater flexibility when thinking about the future for some.

*“I remember in the in the kind of start of recovering when I first got therapy and stuff they would kind of, say, like ‘oh, you might be able to go to Uni in September, you might not be able to do this’. I was supposed to go for summer, I was supposed to be going to Spain with my family for like two months, and they were like ‘you might not be able to do that’ and now there's like, there's nothing in the way of actually going living my life and following like whatever next step, I have, which is incredibly freeing actually thinking about it.”* (P18)

## Recovery representing liberation

All participants with current AN, despite some of them having a chronic course of illness, expressed desire for a life without AN. Some were able to describe what this would entail, which largely centred around feelings of freedom, independence and acceptance. Freedom was described as liberation from the thought processes associated with AN (e.g. fear of food, constant thoughts about food/weight), but also the behavioural characteristics (e.g. inflexibility, lack of spontaneity). Crucially, respondents expressed a desire for improved mood and self-compassion.

“*…so I guess it [recovery] looks like, and it sounds really cheesy, but like, freedom, but just being able to go into a shop and buy anything I want because I fancy it. But also just being able to go out with friends and not worry about food or timings or what I'm going to be eating, or have to look up the menu, and be able to be at home and let my parents cook for me. Yeah, I want to just be able to feel that freedom and like not feel tied down to something as simple as food.”* (P01)

*“I think I would like to be yeah, just comfortable with myself, happier with where I am, more compassionate…”* (P09)

Respondents with current AN were realistic in understanding the necessity of weight gain in order to have the energy and physiological resources for engaging in life and for cognition. Some expressed desire to have a greater flexibility in how they perceive weight gain.

*“And yeah so, for example, if recovery for me and being happy means gaining 10 kilograms, then I hope I'll be fine with this, but if it's 15 then I want to be fine with it as well, and not limiting myself to whatever kind of standard is set in I don't know the NICE guidelines or wherever, um because I think that can be quite negative as well.”* (P08)

The reality of recovery from AN was aligned with these features of increased flexibility and freedom, although additional specific improvements were mentioned by respondents recovered from AN. For example, the alleviation of the intense thoughts surrounding weight/shape/food enabled cognitive resources that could be allocated to aspects of life that were personally meaningful to them. Incidentally, improvements in memory, information processing and concentration were noted, together with less bias in thoughts towards ED-related content.

*“It kind of is in a similar vein to just the very idea that your brain is so taken up with thoughts of food, thoughts of eating, thoughts of calories, thoughts of wanting to be thin and what you ate, what you're going to eat, what you didn't eat. That it's just like your mind doesn't have room for anything else. And I really wanted to have that room, I could recognise I didn't have room for anything else, but I wanted to, and that was really frustrating.”* (P12)

Participants recovered from AN described how they were able to experience a greater spectrum of emotions at a greater intensity, which was in some ways pleasant (e.g. experiencing feelings of joy) but often unpleasant (e.g. experiencing feelings of anger). However, respondents also mentioned having a greater ability to regulate their emotions, particularly those that were negative.

*“I mean it can be tough sometimes, like, you know feel, I’ll like feel really angry, it would be kind of scary because I was so used to being numb. But then I’m able to enjoy things so much more now, like feel happy about things you know if it's like a really nice event or like a wedding, my best friend's wedding was in the summer and I can live a happy moment for me.”* (P19)

Relatedly, improvements in relationships were noted together with feelings of social connectedness. Crucially, some recovered from AN identified achievements resulting from these gains in life as a protective factor against relapsing.

*“Things that I wanted to achieve I feel like that has contributed to like how, yeah, how much now, I think I I don't see myself, I know anorexia- relapse instances are like so common in anorexia but I honestly don't imagine ever going back there...”* (P18)

### Identity as liberation

Those recovered from AN described how liberation from the illness enabled them to engage in activities that gave them a sense of purpose and identity. Participants recovered from AN described being unable to connect with their “ill selves”.

“*I think like one thing I feel like quite strongly about recovery is the like ability to like find out like who... this sounds very like deep and meaningful, but like who like who I am I think that was like. Like what makes me me. Because I think throughout the eating disorder I just completely lost that, like I was overtaken by a completely different personality almost.”* (P17)

Several of the recovered participants described the experience of finding their personal sense of self and belonging as delayed maturity.

*“I feel like I've almost had to learn things that other people learnt when they were like 16 when I was like 22, so, it felt like everything was a little bit delayed.”* (P17)

Notably, recovered participants described their pre-AN self as relaxed, easy-going, and flexible, and described their AN traits as separate from innate characteristics.

*“…my mum has also said when I was younger I was a very relaxed, laid-back child, and I am like now, but in that time, I was like so rigid, so inflexible, and so unable to like adjust...”* (P17)

Although participants currently experiencing AN described feeling consumed by the illness, they were able to describe their pre-morbid interests (e.g. sports, hobbies, vocation, education) and express a desire to regain them again. They often described a strong desire for “living rather than existing” and making a valuable contribution to society.

*“...like it’s always going to be there but being able to replace it with other things so maybe like studying or going to college is really important because it gives my life more meaning that an eating disorder.”* (P02)

## Discussion

This study aimed to explore subjective experiences of cognition and thoughts about the future in the acute stages of AN and following recovery. The use of semi-structured interviews allowed us to identify how cognitive difficulties, many of which have been identified in prior neuropsychological literature, translate to real-life areas of difficulty in people with AN.

Overall, the findings of this study indicate that although people with AN often appear to be high-functioning in the acute stages of illness, there are significant cognitive difficulties that are experienced, which lead to distress, problems with engaging in daily life and may represent barriers to recovery [33]. Many of the findings converge with a prior phenomenological meta-synthesis by Bryant and colleagues [23], although others relating to specific cognitive difficulties build on their findings, most notably in their identified domain of “functional impairment”. Some of the reported cognitive difficulties were features of AN identified in prior research (e.g. poor cognitive flexibility [10], attentional biases; [34–36]), and others were reminiscent of more serious memory difficulties—for example poor visuospatial memory and amnesia of entire life episodes. Respondents described how AN often initially functions as a mechanism for coping with negative affect or stressful life events, but over time hijacks all aspects of life whereby the focus is centred on the demands of the illness. This apparent high level of functioning in daily life (e.g. maintaining a job, succeeding in education) is attributable to the high levels of perfectionism associated with AN, as well as attention and energy being drawn away from social and leisure activities [33]. This inevitably is cognitively demanding; which is an important aspect of the illness to bear in mind when considering people with AN that on the surface appear to be managing quite well despite being acutely unwell. Freedom and liberation were central components of recovery and people who were recovered from AN described the development of a non-AN identity as an important aspect leading to freedom. These findings have important implications for future research investigating cognition in AN and novel interventions.

Poor cognitive flexibility and high levels of rigidity were mentioned as characteristic of AN by all respondents in the present study. These symptoms of AN have been widely explored in previous literature and considered as a potential trait of AN [37]. Several participants reported being generally inflexible before the onset of AN and after recovery, albeit others described inflexibility as being tied to the onset of AN. Moreover, an intriguing link between low flexibility and risk aversion was made by participants. In particular, several respondents described how inflexible behaviour minimised the chances of being in situations where their mood or anxiety could be worsened; inflexibility may be used as an emotion regulation strategy. This aligns with phenomenological findings whereby control and inflexibility represent tools for emotional avoidance and numbing [23], and quantitative findings linking inflexibility to heightened eating disorder symptoms and behaviours [38], in people with AN. In this way, there may be utility in both targeting the low mood and high anxiety associated with AN, formulating interventions in aiding sufferers to experience and regulate emotions as well as to introduce more flexibility [38].

Respondents described difficulty in retrieving details of autobiographical memories, albeit had a better ability for remembering notable (usually negative) events. This aligns with findings of autobiographical memory overgeneralisation in AN [39, 40], as well as the prior neuropsychological study [26], which found that people with AN showed an overgeneralisation when retrieving autobiographical memories, and reported positive memories as less vivid than neutral and negative memories. It is possible this represents a bias in recall in AN, whereby positive memories are less salient in one’s perception of the past. This is contrary to the phenomenon exhibited in non-clinical populations of a “fading affect bias” in memory. This is where negatively-valenced memories fade in intensity over time but positively-valenced memories do not, which is thought to maintain psychological wellbeing [41]. Following the constructive simulation hypothesis, which posits that the construction of future events relies on the flexible recombination of specific details retrieved from autobiographical memories [42], the simulation of future events would likewise obtain a negative bias. Indeed, in the present study respondents with AN had difficulty imagining the future, or were heavily biased towards AN, even if they had periods of recovery in the past. On the other hand, those recovered from AN reported that living away from the constraints and demands of AN allowed them to think more flexibly about the future.

Notably, there were several reasons proposed for broad memory difficulties in AN. Respondents acknowledged the impact of starvation on their wider brain function and subsequent cognitive processes, but also emphasised the importance of attentional bias on information processing and memory encoding. It is possible that there are converging aspects of AN that result in memory problems. Firstly, the physical sequelae of starvation, including reductions in hippocampal volume [4] and growth factors such as brain-derived neurotrophic factor [43], are likely to contribute to difficulties in memory consolidation and storage [44, 45]. Secondly, the high level of attentional bias and sustained control towards food-related information, may reduce cognitive resources and interfere with information processing and subsequent learning and memory encoding [15, 46]. Thirdly, the generalised problems with information processing associated with fatigue, “brain-fog”, and poor concentration, due to low weight, may also interfere with attention, information processing and subsequent learning and memory encoding [47]. Whilst the first suggestion cannot be supported by the methodology and findings of the present study, the second and third suggestions can be somewhat facilitated (albeit not proven) by the poor information processing and concentration, attentional bias towards AN content, and fatigue reported by respondents. Extended duration of illness may worsen this process; those with longer illness duration (> 9 years) in the current study reported noticing their memory and wider cognitive problems worsening over the course of the illness, aligning with other phenomenological findings [23].

Respondents with longer illness duration appeared to have incorporated AN more extensively into their identity; over time, the non-AN identity became more elusive and unlikely. The cognitive and affective aspects of AN described by respondents in the present study may disrupt both an individual’s ability (cognitive resources) and motivation to recover. However, the development of aspects of identity that are separate from AN, thus replacing the egosyntonic features of AN [48], was identified as a core aspect of recovery by those who had recovered. These findings reflect prior phenomenological evidence of the inextricable bind between AN and one’s identity [23]. The reduced ability to retrieve autobiographical memories may disrupt an individual’s ability to form a self-narrative; the story of an individual as experienced through their own eyes and experience. The formation of a strong self-narrative is crucial for the understanding of one’s identity [49]. Thus, this is a pathway in which impaired autobiographical memory retrieval could act as a barrier to recovery. The formation of a rich non-AN identity may sustain recovery. An influential study published in 1987 by Patricia Linville outlined how people with more complex self-concepts had higher levels of resilience in the context of stress-related illness and depression, as failure in one domain doesn’t implicate the entire self-concept [50]. Respondents in our study reported how AN behaviours are used as a coping mechanism for difficult emotions. Therefore, the formation of a complex self-identity may allow individuals with AN to draw upon other aspects of their self-concepts when facing difficult emotions rather than resorting to AN behaviours. In summary, it is important to understand how problems with memory retrieval and/or biases in memory recall may feed into the nature of self-narratives and what barriers this may present for engaging in and sustaining recovery.

### Clinical implications and future research

The insights obtained from interviewing people with AN inspire several potential research avenues. Firstly, the present study highlights the importance and value in investigating the phenomenology of AN from both acute and recovered perspectives. The high degree of socio-occupational functioning, high detail-focus and presence of perfectionist traits in people with AN obscures the extent to which cognitive difficulties (e.g. those highlighted in this study) impact on their daily function and quality of life. These findings build on our understanding of, and contextualise, functional impairments experienced by people with AN that have been identified by prior phenomenological research [23]. Therefore, we recommend that further research take similar approaches to investigate cognition in AN, using qualitative methodologies. Phenomenological investigations, especially of subjective experiences that may be concealed by the person with AN, may also have utility in providing clinicians, carers, family, partners and friends with greater insight into the role and impact of AN.

Pertaining to biological treatments, recombinant human leptin (metreleptin) has been used in some preliminary case studies to successfully ameliorate some of the cognitive, emotional and behavioural symptoms associated with AN [51, 52] that are reported in the present study without causing weight loss (see [53] for a comprehensive review of hypoleptinemia and the use of metreleptin in AN). In the context of psychological therapies, the present study points towards several potential future avenues. For example, the importance of freedom and flexibility in life following recovery indicates support for neuropsychologically-based interventions such as cognitive remediation therapy, which has already shown promise as an adjunctive treatment for AN [54]. Moreover, it is apparent that developing identity is a vital component of recovery from AN, albeit represents a particular challenge given the strong sense of identity and achievement that AN itself provides [23]. The Maudsley Model of Anorexia Nervosa Treatment for Adults (MANTRA) [55], a specialised integrative therapy for AN, has identity development as a core module. Further interventions centred around the development of identity may be useful, such as narrative-based therapies or social prescribing, whereby individuals are encouraged to engage with their local communities as well as artistic and cultural experiences [56].

It has been recommended that clinicians, and further research, should attend to the mental capacity of people with AN, particularly in protracted cases [57]. Mental capacity is a medical-legal term. In the UK, for example, it is defined in Sect. 3 of the Mental Capacity Act 2005 as the ability to make a decision which includes the ability to understand the information relevant to the decision, retain that information, use or weigh up that information as part of making the decision, and communicate the decision [58]. The assessment of mental capacity has serious implications in a medical-legal context and in particular surrounding the consent to treatment. This is particularly the case in the context of severe and enduring AN, where amnestic and cognitive difficulties associated with low weight may be more advanced and long-standing, and complex decisions regarding clinical management are likely to ensue.

### Limitations

There are a number of limitations to the current study. Firstly, the sample used in the study were all adults, limiting its representativeness; a high proportion of people with AN are in their adolescence [59] and it is possible that cognitive difficulties are different in this developmental stage [60]. The duration of recovery in the recovered group was relatively low and the recall of experiences of AN in individuals with a greater duration of recovery is likely to be different. However, it is interesting that despite this relatively short period of recovery, many respondents reported an inability to remember their period of illness. Moreover, all respondents had previously taken part in a neuropsychological study investigating autobiographical memory and episodic future thinking [26], where they were fully debriefed. Therefore, all respondents had some prior knowledge of the overall research objectives, which may have biased their responses. However, one strength of qualitative methods is the opportunity to obtain rich information on a specific topic, grounded in individual experiences, whereby disclosure of that topic is necessary. Finally, given that people with AN tend to experience alexithymia (problems in feeling and labelling emotions) [61] and poor interoception (internal body state awareness) [62], it is likely that the AN group found it particularly difficult to reflect on their own cognitive and emotional state. This may explain why many of the cognitive difficulties associated with AN in the present study were reported in retrospect by those in the recovered AN group, when alexithymia and poor interoception may improve [63, 64].

### Conclusions

This study endeavoured to explore the experiences of cognition within AN and following recovery, through semi-structured interviews of individuals currently living with AN and those who have recovered. Several cognitive and emotional difficulties (e.g. poor cognitive flexibility and autobiographical memory retrieval, alexithymia) aligning with prior literature were reported as features of the acute experience of AN. Possible links between these cognitive difficulties were proposed, as well as features of the illness as explanatory factors, such as the physical effects of starvation. Individuals with AN often seem to function remarkably well in their academic and vocational lives, giving the impression of little impairment. However, the respondents in the present study described how the cognitive and emotional difficulties cause significant distress and interfere with their engagement in daily life, reducing overall quality of life. On the other hand, recovery was described as liberation from these cognitive difficulties, with an emphasis on the development of a non-AN identity and the development of tools to better manage negative emotions and stress.

## Supplementary Information

**Additional file 1**. Supplementary Materials.

## Data Availability

The datasets used in the current study are available from the corresponding author on reasonable request.

## Abbreviations

AN: Anorexia Nervosa
AN-BP: AN Binge-Purge subtype
AN-R: AN Restrictive subtype
ASD: Autism Spectrum Disorder
BMI: Body Mass Index
DASS: Depression, Anxiety and Stress Scale
EDE-Q: Eating Disorder Examination-Questionnaire
EUPD: Emotionally Unstable Personality Disorder
M: Mean
PTSD: Post-Traumatic Stress Disorder
Rec-AN: Recovered from AN
SD: Standard deviation
UK: United Kingdom
