# Supplementary material for: “Your mind doesn’t have room for anything else”: a qualitative study of perceptions of cognitive functioning during and after recovery from anorexia nervosa
Source: J Eat Disord. 2022 Dec 27;10:201. doi: 10.1186/s40337-022-00723-6 (PMC9793561; doi:10.1186/s40337-022-00723-6)
Supplement: Supplementary file 1 — Additional file 1. Supplementary Materials. [file 40337_2022_723_MOESM1_ESM.docx]

Supplementary Materials for “’Your mind doesn’t have room for anything else’: a qualitative study of perceptions of cognitive functioning during and after recovery from anorexia nervosa”: Semi-structured interview schedule

**Section 1: Background and general cognitive function**

1. “What are your experiences of living with anorexia nervosa?”
2. “How has anorexia impacted on your ability to function in everyday life?”
3. “How has anorexia affected the way that you think/your thinking processes?”
   1. “Specifically, did you notice any changes in the way that you remembered information or events? If so, please could you give some more details?”
   2. “What would you attribute this to?”
4. “Can you remember a specific incident associated with your eating disorder?”
5. “Can you speak more about the period of your eating disorder more generally?”
   1. “How vividly do you recall this?”

**Section 2: Change over time**

1. (RecAN only) “Thinking back to when you had AN and now, have you noticed any changes?” (Prompts relating to cognitive aspects)
2. (AcAN only) “Thinking back to when you first developed symptoms of AN and now, have you noticed any changes?” (Prompts relating to cognitive aspects)

**Section 3: Thinking about the future**

1. (AcAN only) What does recovery look like to you?
2. “Imagine yourself in 10 years time. Where will you be, and what will you be doing?”
   1. If reporting that they can’t imagine a future – “why do you think that may be?”
3. “Did you find thinking about yourself in 10 years time an easy task?”
